# Performance and Health Benefits of Dietary Nitrate Supplementation in Older Adults: A Systematic Review

**DOI:** 10.3390/nu9111171

**Published:** 2017-10-27

**Authors:** Luke Stanaway, Kay Rutherfurd-Markwick, Rachel Page, Ajmol Ali

**Affiliations:** 1 School of Sport, Exercise and Nutrition, Massey University, Auckland 0745, New Zealand; luke_ruawai@hotmail.co.nz; 2 School of Health Sciences, Massey University, Auckland 0745, New Zealand; K.J.Rutherfurd@massey.ac.nz; 3 Centre for Metabolic Health Research, Massey University, Auckland 0745, New Zealand; R.A.Page@massey.ac.nz; 4 School of Health Sciences, Massey University, Wellington 6140, New Zealand

**Keywords:** nitric oxide, beetroot juice, older adults, cognition, cardiovascular, blood pressure

## Abstract

Supplementation with nitrate (NO_3_^−^)-rich beetroot juice has been shown to improve exercise performance and cardiovascular (CV) responses, due to an increased nitric oxide (NO) availability. However, it is unclear whether these benefits are greater in older adults who have an age-related decrease in NO and higher risk of disease. This systematic review examines 12 randomised, crossover, control trials, investigating food-based NO_3_^−^ supplementation in older adults and its potential benefits on physiological and cognitive performances, and CV, cerebrovascular and metabolic health. Four studies found improvements in physiological performance (time to exhaustion) following dietary NO_3_^−^ supplementation in older adults. Benefits on cognitive performance were unclear. Six studies reported improvements in CV health (blood pressure and blood flow), while six found no improvement. One study showed improvements in cerebrovascular health and two found no improvement in metabolic health. The current literature indicates positive effects of dietary NO_3_^−^ supplementation in older adults on physiological performance, with some evidence indicating benefits on cardiovascular and cerebrovascular health. Effects on cognitive performance were mixed and studies on metabolic health indicated no benefit. However, there has been limited research conducted on the effects of dietary NO_3_^−^ supplementation in older adults, thus, further study, utilising a randomised, double-blind, control trial design, is warranted.

## 1. Introduction

Over the past decade, there has been a decrease in levels of functional activity (physiological performance) and an increase in frailty, in the aging population, alongside increased rates of disease and age-related dysfunction [1,2]. An individual’s performance and health in their later years of life can be influenced by many lifestyle factors; some are more controllable and have greater impact on performance and health-related outcomes than others [2]. For example, dietary habits have been indicated as a dominant influencer of healthy aging and age-related dysfunction, which can be easily improved to promote beneficial outcomes [3]. Due to this significant influence of diet, recent research has begun to focus on food-based supplements, which contain key bio-active compounds, proposed to promote performance and health benefits [4].

One key nutritional ingredient that has gained recent attention is inorganic nitrate (NO_3_^−^). Nitrate is found in high concentrations in green leafy vegetables, such as spinach and rocket, and root vegetables such as beetroot [2]. Additionally, nitrate is a precursor for nitric oxide (NO)—the bio-active form of nitrate—which has many functions in the human body, ranging from regulation of neurotransmission, immunity and blood flow, to alterations in oxygen consumption, and has been shown to have many performance (both physiological and cognitive) [5,6] and health benefits [7]. It is important to note that an acceptable daily intake of nitrate has been set at 3.7 mg·kg^−1^, as a small percentage of nitrate is also converted to N-nitrosamines in the human body, which have been implicated in certain cancers, such as gastric, pancreatic and thyroid cancers [4]. Negative side effects are commonly associated with consumption of nitrate salts, such as those found in cured meats [2,4]. Despite this, there is currently no evidence to indicate negative effects of prolonged nitrate consumption from vegetable sources, such as beetroot [2,4].

There are two known pathways for NO production in the human body. The first is the endogenous pathway, where l-arginine is converted to NO by nitric oxide synthases (NOS), which until recently was believed to be the sole pathway [4,8]. The second pathway is the exogenous pathway. Following consumption of dietary NO_3_^−^, it is rapidly absorbed from the stomach and duodenum of the small intestine and enters the blood stream [6]. About 20–25% of the NO_3_^−^ is absorbed from the circulation and concentrated (10 to 20-fold) in the salivary glands [9]. Facultative anaerobes, located in the crypts on the dorsal surface of the tongue, reduce NO_3_^−^ to the bioactive nitrite (NO_2_^−^), which is swallowed again [4]. Small amounts of this NO_2_^−^ will be reduced to NO in the acidic environment of the stomach, while most re-enters the systemic circulation and is transported to specific locations in the body, where it can be reduced to NO, via one or more potential non-enzymatic or enzymatic pathways [10]. The exact mechanism behind how NO^2−^ enters the circulation and is reduced is still unclear; however, this reduction tends to occur in hypoxic tissue and conditions of low pH [4,10]. This reduction occurs predominately in the vasculature [11]. It is also important to note that through this NO^3^–NO^2^–NO pathway, endogenous NO^3−^ can be recycled by oral bacteria to act as a reservoir of precursors for NO production and that interruption of this recycling (such as from use of antiseptic mouthwash) has been shown to decrease plasma nitrite levels and raise blood pressure (BP) in individuals in the absence of nitrate intake [4,10].

The ability for consumption of dietary NO_3_^−^ to result in an increased production of NO, via the aforementioned exogenous pathway, has led to greater interest in dietary NO_3_^−^ (particularly in the form of beetroot juice), as a food-based supplement with the potential to improve performance and health [2]. Furthermore, it has been shown that NO production in the body decreases with age and is believed to be a contributor to age-related dysfunction and increased risk of disease [4]. Therefore, more recently, specific interest has been directed at the benefits of NO_3_^−^ supplementation in the older population.

Previous studies have shown that both acute and prolonged supplementation of dietary NO_3_^−^ in older adults can significantly improve oxygen uptake ($\dot{V}O_{2}$) responses during exercise [8], and increase time to fatigue [5,12,13], thus promoting improved exercise performance. Furthermore, it has also been shown that NO_3_^−^ supplementation can significantly improve cognitive performance, as shown by enhanced reaction times [6] in older adults. However, the investigation into cognitive performance benefits with dietary NO_3_^−^ supplementation is scarce, with equivocal findings [8]. In addition to the potential performance benefits, studies have shown positive health-related outcomes in older adults, with acute and prolonged dietary NO_3_^−^ supplementation, including reduced blood pressure [7,12] and improved blood flow to the muscle and brain [13,14], indicative of potential cardiovascular and cerebrovascular benefits, which may result in a lower risk of certain diseases. As older adults have age-related diminishments in functional capacity of epithelial vasodilation (reducing blood flow), cardiovascular function, cognitive function, and mood, there is greater potential for improvements in these areas with NO_3_^−^ supplementation, relative to younger adults who generally possess optimal functional capabilities [13].

Therefore, the purpose of this systematic review was to critically evaluate the effects of dietary NO_3_^−^ supplementation on performance (physiological and cognitive) and health factors (specifically, cardiovascular, cerebrovascular and metabolic health) in older adults. This is the first systematic review of its kind, evaluating potential benefits of dietary NO_3_^−^ supplementation, specifically in older adults.

## 2. Materials and Methods

A systematic literature search was conducted by one researcher (L.S.) to identify primary research in the area of inorganic dietary NO_3_^−^ supplementation in older adults, with the aim of examining performance and health benefits associated with dietary NO_3_^−^ consumption.

### 2.1. Types of Outcome Measures

The primary focuses of this systematic review were any performance and health parameters affected by dietary NO_3_^−^ supplementation in older adults. This included changes in physiological and cognitive performance, as well as cardiovascular, cerebrovascular, metabolic, and bone health.

### 2.2. Search Method

The following online databases were systematically searched: PubMed, Ovid, Science Direct and Web of Science, from inception to May 2017. The keywords chosen for the search included: nitrate OR nitrates OR beetroot AND older adults OR elderly. This search was enhanced via manual examination of the reference lists of all retrieved papers in attempts to detect any other potentially eligible studies.

### 2.3. Inclusion and Exclusion Criteria

The inclusion and exclusion criteria for all retrieved papers were established by the researchers (L.S., A.A., K.R. and R.P.). This systematic review only included primary research published in peer-reviewed journals, in English, using a randomised, crossover, placebo-controlled design. In addition, studies had to meet a set criteria including: (1) participants had to be older adults (aged 50+) or involve older adults in the study as a separate age group, i.e., those comparing younger with older adults; (2) participants could be healthy or possess one or more morbidities (e.g., hypertension, diabetes, peripheral arterial diseases); (3) studies had to investigate the effects of inorganic dietary NO_3_^−^ supplementation (such as beetroot juice or high nitrate diet interventions) on performance and/or health parameters; (4) studies using multiple supplementation protocols, involving other supplements in addition to nitrate, had to show a clear separation in the effects of NO_3_^−^ and the other supplements.

### 2.4. Study Selection

**Figure 1:**
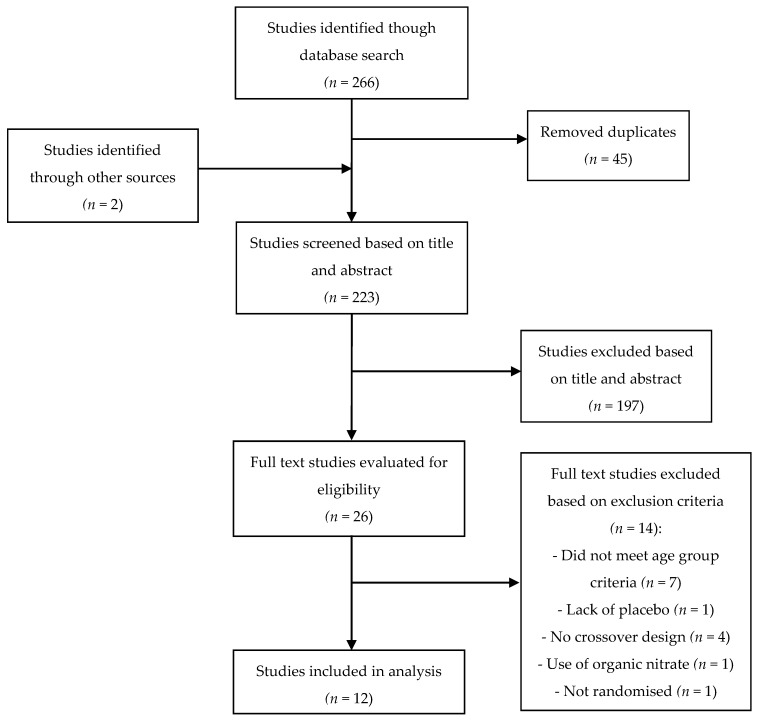
Flowchart showing study selection procedure and results.

Following the removal of duplicate studies from the different search engines, independent screening of the title and abstract of the remaining articles was undertaken to determine eligibility. Selected papers were then read in full to finalise eligibility. A summary of this process is outlined in Figure 1.

### 2.5. Quality Assessment

Risk of bias was assessed informally, using a bias hierarchy checklist, as described by Wright et al. [15], including the following assessment of key areas of bias: selection, performance, detection and attrition bias. Each article was individually assessed by the reviewer, L.S.

### 2.6. Data Extraction

Data were extracted independently by one of the researchers (L.S.) and reviewed by the other researchers (A.A., K.R. and R.P.), following a systematic selection list. This included: (1) participant data (number of participants, sex distribution, health stasis (i.e., healthy or diagnosed patients), and age); (2) study design; (3) supplementation protocol; (4) testing protocol used; and (5) primary outcome measures (physiological performance, cognitive function, and health-based parameter). Mean values were extracted for all data, where appropriate.

## 3. Results

Twelve studies [5,6,7,8,12,13,14,16,17,18,19,20] out of a total of 266 studies identified from preliminary research of online databases and other sources after reference list screening, were included in this systematic review (Figure 1).

### 3.1. Participant Characteristics

A summary of the characteristics of the studies is outlined in Table 1. From the 12 included studies, there were a total of 202 participants (95 males, 93 females, and 14 not stated). The number of participants in each trial ranged from 8 [13,18] to 27 [6,17], with mean ages ranging from 59.2 [19] to 74.7 years [14]. These 12 studies targeted older adult participants of varying health status. Five studies recruited healthy older adults [8,14,18,19,20], two studies recruited older adults with type 2 diabetes [6,17], one study recruited participants with chronic obstructive pulmonary disease (COPD) [5], one recruited participants with heart failure with preserved ejection function (HFpEF) [12], one with peripheral arterial disease (PAD) [13], one with chronic kidney disease (CKD) [7] and one with risk factors for cardiovascular disease [16].

### 3.2. Study Characteristics

All studies implemented a randomised, crossover design, involving a placebo treatment and at least one NO_3_^−^ treatment. Of the 12 studies, six used a NO_3_^−^ depleted beetroot juice as the placebo [6,8,12,17,19,20], two used a low NO_3_^−^ diet [14,18], and single studies used NO_3_^−^ depleted gel [16], water [7], prune juice [5], or orange juice [13]. Seven studies were double-blinded [6,8,12,16,17,19,20], one was single-blinded [5], and four were open-label trials [7,13,14,18].

### 3.3. Study Quality

Based on an informal assessment of bias, the studies included in this systemic review were considered to be good to excellent, based on the above-mentioned study characteristics. All studies were placebo controlled, randomised crossover experiments (low selection/detection bias), with the majority being double- or single-blinded (low performance/detection bias).

### 3.4. Nitrate Supplementation

The majority of the studies (*n* = 10) utilised supplemented inorganic NO_3_^−^ in the form of beetroot juice, while the remaining two studies used beetroot gel and a high NO_3_^−^ diet, where beetroot juice was included in the diet. The difference in the NO_3_^−^ dose used between studies ranged from 6.1 to 12.4 mmol·day^−1^ (approx. 350–700 mg·day^−1^). In addition, the supplementation period varied between studies: five studies investigated acute supplementation (consumption 2–4 h prior to testing [5,7,13,16,19]), six investigated chronic supplementation (2.5–14 days of NO_3_^−^ loading [6,8,14,17,18,20]), and one study investigated both acute (1.5–2 h prior) and chronic (7 days) supplementation [12].

### 3.5. Adverse Effects of Supplementation

Five of the 12 studies [14,15,16,17,18,20] reported minor adverse effects from dietary NO_3_^−^ supplementation; two studies reported beeturia (red urine) [16,20], two studies reported both beeturia and red stools [14,17], and one study reported beeturia, gastrointestinal discomfort and headaches [18]. No chronic adverse effects from NO_3_^−^ supplementation were stated in any of the 12 studies.

### 3.6. Characteristics of Nitric Oxide Measures

The 12 studies incorporated various measures to investigate the potential changes in NO concentrations in the body following supplementation with dietary NO_3_^−^, including plasma [NO_3_^−^] and [NO_2_^−^], and urinary [NO_3_^−^] and [NO_2_^−^] measures. Seven studies reported both plasma [NO_3_^−^] and [NO_2_^−^] [5,6,12,13,14,17,18,19], one measured solely plasma [NO_2_^−^] [8], one measured solely plasma [NO_3_^−^] [7], one measured both plasma and urinary [NO_3_^−^] [20], and one measured both urinary [NO_3_^−^] and [NO_2_^−^] [16]. Similarly, various methods were used to determine the aforementioned values. Five studies used a Sievers NO analyser (chemiluminescence-based) [6,13,14,17,19]; three used an ENO-20 NO analyser (based on colorimetric Griess assay) [5,12,18]; one used high-performance liquid chromatography (HPLC) [16]; one used gas chromatography-mass spectroscopy (GC-MS) [20]; one used an enzyme-linked immunosorbent assay [7]; and one used a modified chemiluminescence method [8].

### 3.7. Outcomes

#### 3.7.1. Nitric Oxide Indices

Despite the different NO indices used between studies, dietary NO_3_^−^ supplementation resulted in a significant increase in all NO indices (compared to placebo) in all 12 studies. Specifically, increases in plasma [NO_3_^−^] ranged from 150% [20] to 938% [5], while increases in plasma [NO_2_^−^] ranged from 168% [17] to 503% [8] and increases in urinary [NO_3_^−^] were 283% [16] and 979% [20], while the increase in urinary [NO_2_^−^] was 214% [16], at pre- versus post-supplementation, with dietary NO_3_^−^.

#### 3.7.2. Performance Outcomes

##### Physiological Performance

Five of the 12 studies investigated the effects of dietary NO_3_^−^ supplementation on physiological performance in older adults [5,8,12,13,20]. Acute supplementation with dietary NO_3_^−^ (7.58 mmol/400 mg NO_3_^−^), 2.5 h prior to exercise, significantly improved time to exhaustion during submaximal cycling (*p* = 0.031) [5]. Likewise, 7 days of supplementation with NO_3_^−^ rich beetroot juice (6.1 mmol·day^−1^/350 mg NO_3_^−^) also improved time to exhaustion during submaximal cycling exercise (*p* = 0.02) [12]; however, this improvement was not seen during the acute supplementation protocol in the study. Additionally, acute supplementation with NO_3_^−^ rich beetroot juice (9 mmol/500 mg NO_3_^−^) improved walking duration prior to onset of pain by 18%, and maximal walking time by 17% in older adults with chronic obstructive pulmonary disease (COPD) [13]. Similarly, an increase in the $\dot{V}O_{2}$ response time from rest to moderate intensity walking (*p* < 0.05) was shown, following 3 days of supplementation with beetroot juice (9.6 mmol·day^−1^/550 mg NO_3_^−^) [8]. Despite this, the same study found no change in a 6-min walking test [8]. Additionally, it was reported that 7 days of supplementation with NO_3_^−^ rich beetroot juice (12 mmol·day^−1^/700 mg NO_3_^−^) had no effect on O_2_ consumption, a 10-m walking test, a hand-grip strength test, an up-and-go test, or a repeated chair raising test, in healthy older adults [20].

##### Cognitive Performance

Of the 12 studies, only three investigated the effects of dietary NO_3_^−^ supplementation on cognitive performance in older adults [6,8,14], with only two directly measuring cognitive function [6,8]. The potential for dietary NO_3_^−^ as a cognitive performance aid in older adults consuming a high NO_3_^−^ diet, including beetroot juice (total NO_3_^−^ ~12.4 mmol/700 mg), for 2 days, was shown by an increase in cerebral blood flow to the white matter of the frontal lobe (*p* < 0.005) [14]. Additionally, 14 days of supplementation with NO_3_^−^ rich beetroot juice (250 mL·day^−1^, ~7.5 mmol/400 mg NO_3_^−^) in older adults (67.2 ± 4.9 years) with type 2 diabetes, resulted in a significant improvement in simple reaction time, compared to a placebo [6]. Despite this, there was no difference between treatments for other cognitive tests, including decision time, rapid processing, and spatial and shape memory [6]. Investigation of the effects of NO_3_^−^ rich beetroot juice supplementation over 3 days (2 × 70 mL·day^−1^, ~9.6 mmol/550 mg of NO_3_^−^) on cognitive performance, in healthy, older adults (aged 60–70), showed no significant differences in memory, attention and information processing capabilities between the beetroot juice and placebo treatments [8].

#### 3.7.3. Health Outcomes

##### Cardiovascular Health

Ten studies investigated the potential benefits of dietary NO_3_^−^ supplementation on BP in older adults. Five of these studies showed that dietary NO_3_^−^ supplementation resulted in a significant decrease in systolic blood pressure (SBP) [5,7,8,12,13], with four of the five studies also showing a significant decrease in diastolic blood pressure (DBP) following NO_3_^−^ supplementation, compared to a placebo [5,7,8,13]. In contrast, five studies found no change in the BP of older adults after supplementation with dietary NO_3_^−^ [16,17,18,19,20]. There were two studies that did not measure changes in BP [6,14]. Furthermore, two studies also investigated mean arterial pressure (MAP) (the average pressure in arteries from one cardiac cycle) following beetroot juice supplementation. Gilchrist et al. [17] found no difference between treatments (BR, 7.5 mmol/400 mg NO_3_^−^ vs. nitrate-depleted PL) in older adults with type 2 diabetes, whereas Kemmner et al. [7] showed a decrease in MAP in older adults with CKD, following acute supplementation with NO_3_^−^ rich beetroot juice (200 ml, 6 mmol/350 mg NO_3_^−^) (*p* = 0.012) [7]. Three studies investigated endothelial function and blood flow in older adults in response to dietary NO_3_^−^ supplementation [13,16,17]. Acute beetroot juice supplementation in older adults (67 ± 13 years) with PAD was found to increase blood flow to the working muscles, using near infrared spectroscopy (NIRS) [13]. Acute supplementation with 100 g of NO_3_^−^ rich beetroot gel (12.2 mmol/700 mg NO_3_^−^) in older adults with risk factors of cardiovascular disease has also been reported to significantly improve brachial flow-mediated dilation (FMD) (77%), blood flow velocity (BFV) (31%), and reactive hyperaemia (RH) (18%), thus improving endothelial function [16]. However, 14 days of supplementation with NO_3_^−^ rich beetroot juice in older adults with type 2 diabetes had no significant effect on FMD or Doppler perfusion, and thus, did not improve endothelial function [17].

The study by Kemmner et al. [7] also reported a decrease in the renal resistance index (RRI) (*p* = 0.017) following acute supplementation with NO_3_^−^ rich beetroot juice in older adults with CKD.

##### Cerebrovascular Health

Only one study has investigated the potential cerebrovascular health benefits of dietary NO_3_^−^ supplementation in older adults [14]. This study used magnetic resonance imagining (MRI) to show increased perfusion in the frontal cortex of the brain (*p* < 0.005) of older adults following 2 days of a high NO_3_^−^ diet (including beetroot juice), compared to a diet low in NO_3_^−^ [14].

##### Metabolic Health

Of the 12 studies, two investigated various metabolic health parameters following dietary NO_3_^−^ supplementation in older adults [17,19]. Chronic supplementation with NO_3_^−^ rich beetroot juice in older adults with type 2 diabetes had no significant effect on insulin sensitivity, measured via the hyperinsulinemic isoglycemic clamp technique [17]. Furthermore, acute supplementation with NO_3_^−^ rich beetroot juice was also shown to have no significant effect on acute plasma glucose, hepatic blood flow, C-peptide concentration, or incretin concentration (glucagon-like peptide-1 (GLP-1)), in older adults with type 2 diabetes [19].

## 4. Discussion

This systematic review investigated the effects of NO_3_^−^ supplementation on performance and health-related outcomes in older adults. The current literature indicates positive effects of dietary NO_3_^−^ supplementation in older adults on physiological performance, with some evidence indicating benefits on cardiovascular and cerebrovascular health. Effects on cognitive performance were mixed and studies on metabolic health indicated no benefit.

### 4.1. Nitrate Supplementation and Physiological Performance

Dietary NO_3_^−^ supplementation has the potential to improve physiological performance, by prolonging time to exhaustion and increasing the $\dot{V}O_{2}$ response time in older adults. Four of the five studies that investigated physiological performance in older adults found positive outcomes in time to exhaustion during submaximal exercise [5,12,13], and in $\dot{V}O_{2}$ response time [8], while only one study showed no significant effects on physiological performance parameters [20]. This is a similar finding to the systematic review and meta-analysis conducted by Hoon et al. [21] on the effects of dietary NO_3_^−^ supplementation in exercise performance of healthy, young individuals, who reported a pooled effect size of 0.79 for time to exhaustion trials, with favourable benefits from beetroot juice supplementation.

Two studies reported an increase in total exercise capacity following acute supplementation of NO_3_^−^ rich beetroot juice, during submaximal cycling and walking exercise in older adults with COPD and PAD, respectively [5,13]. In contrast, another study found no significant effect of acute beetroot juice supplementation on time to exhaustion during submaximal cycling exercise in older adults with HFpEF [12]. The disparity in findings between these studies may have been due to variances in study design. The two studies which reported improvements in time to exhaustion used an open-label design, where the drinks for the control trials were prune juice [5] and orange juice [13], which could have produced a ‘placebo’ effect; however, Eggebeen et al. [12] used a double-blind design with NO_3_^−^ depleted beetroot juice as the control. Moreover, Berry et al. [5] and Kenjale et al. [13] also utilised higher dosages of NO_3_^−^ and longer absorption periods (7.58 mmol NO_3_^−^, 2.5 h prior, and 9 mmol NO_3_^−^, 3 h prior, respectively), while Eggebeen et al. [12] had participants consume 6.1 mmol NO_3_^−^, 1.5–2 h prior to exercise. These reductions in dosage and absorption times could have prevented adequate concentration and/or time for the supplementation to elicit an effect, especially when, in the same study by Eggebeen et al. [12], while no acute effect was seen, they did show an increased time to exhaustion, after 7 days of dietary NO_3_^−^ supplementation. Nevertheless, another study showed no effect of 7 days supplementation (12 mmol·day^−1^ NO_3_^−^) on time to exhaustion during an incremental cycle test [20]; thus, the discrepancies between the studies could be due to other factors.

Siervo et al. [20] also found no significant improvements in O_2_ consumption, grip strength, up-and-go test, repeated chair raises, or 10-m walking time. However, upon closer examination of the results there was a trend (*p* = 0.10) for improved time to exhaustion and an improvement in all tests, following beetroot versus placebo treatment [20]. This may be a result of insufficient sample sizes to portray the full effects (*n* = 19). Additionally, the absence of significant differences in this study may have been due to the use of healthy older adults, where the aforementioned studies who showed improvements utilised COPD [5], PAD [13], and HFpEF [12] patients who already had diminished physiological functions, and thus had a greater potential to benefit from dietary NO_3_^−^ supplementation [4].

The aforementioned improvements in time to fatigue following NO_3_^−^ supplementation may be due to enhanced blood flow to the working muscle, improvements in mitochondrial efficiency, and/or improved muscle contractile function [22]. There is strong evidence to suggest that supplementation of dietary NO_3_^−^ results in a significant reduction in both systolic and diastolic blood pressure in older adults, which is indicative of increased vasodilation and therefore enhanced blood flow [7,8]. Kenjale et al. [13] showed that, alongside the physiological performance benefits of beetroot juice supplementation, participants also experienced significant drops in DBP, SBP and heart rate (HR), indicating NO-mediated vasodilation and increased blood flow to the working muscles. Near infrared spectroscopy (NIRS) also showed increased muscle tissue oxygenation following supplementation with NO_3_^−^ rich beetroot juice [13]. Increased blood flow to the working muscle allows for greater transport of oxygen, which can result in more efficient metabolic control and prolonged time to fatigue at a given workload, thus improving physiological performance [22].

Furthermore, NO_3_^−^ rich beetroot juice has been shown to improve mitochondrial efficiency via up-regulation of the protein, adenosine nucleotide translocase (ANT), which is involved in the control of flow of mitochondrial protons, resulting in a reduction in proton leakage and increased efficiency of oxidative phosphorylation [23]. In addition, dietary NO_3_^−^ supplementation increased the mitochondrial phosphate to oxygen (P/O) ratio (the amount of ATP produced from a given amount of oxygen reduced), thus reducing the amount of O_2_ required to maintain a given workload and prolonging time to exhaustion [23].

Bailey et al. [24] used ^31^P-magnetic resonance spectroscopy to show a decrease in utilisation of skeletal muscle phosphocreatine (PCr), along with a decrease in production of adenosine diphosphate (ADP) and inorganic phosphate (P_i_), indicating a reduction in the rate of ATP consumption following consumption of NO_3_^−^ rich beetroot juice. This effect indicated that supplementation with dietary NO_3_^−^ (via NO mediated effects) may alter the ATP consumption rates of actomyosin-ATPase and Ca^2+^-ATPase during muscle contraction [24]. Therefore, the reductions in ATP utilisation from these two ATPases during contraction would result in the ability to perform a given work rate for longer, thus prolonging time to exhaustion [25].

Collectively, the results indicate that dietary NO_3_^−^ supplementation can result in improved blood flow and mitochondrial efficiency, and a reduced rate of ATP utilisation, leading to a prolonged time to exhaustion during submaximal exercise [22]. It is important to note that some of the results were based on studies using younger adults [23,24,25]; however, it has been suggested that the same underlying mechanisms would be present in the older population [2]. Additionally, this emphasises the need for further research into the effects on dietary NO_3_^−^ supplementation in older adults.

### 4.2. Nitrate Supplementation and Cognitive Performance

Older adults show reductions in cerebral blood flow (CBF), which may be due to an age-related decrease in the production of NO [4]. This reduction in blood flow to the brain has been indicated as a major risk factor for the impairment of cognitive function and development of neurodegenerative diseases, such as dementia [8]. Therefore, supplementation with NO_3_^−^ rich beetroot juice could have greater benefits on cognitive function in older adults, via its NO mediated increase in CBF [14]. However, in comparison to the effects of dietary NO_3_^−^ supplementation on physiological performance, the effects on cognitive performance in older adults have only just begun to be investigated, thus the effects are less clear [4].

Only two of the 12 studies directly measured the effects of dietary NO_3_^−^ supplementation on cognitive performance in older adults [6,8]. One study showed an improvement in cognitive performance, via a reduction in simple reaction time, with beetroot juice consumption compared to placebo, following a chronic dosing protocol (14 days of NO_3_^−^ rich beetroot juice, 250 mL·day^−1^, ~7.5 mmol NO_3_^−^), in type 2 diabetic, older adults (67.2 ± 4.9 years) [6]. However, they found that performances in decision time, rapid processing, spatial and shape memory tests were not significantly different. The earlier study by Kelly et al. [8] found no effect of dietary NO_3_^−^ supplementation on cognitive performance following 3 days of supplementation with beetroot juice (2 × 70 mL·day^−1^, ~9.6 mmol of NO_3_^−^) in healthy, older adults (males 64 ± 4 and females 63 ± 2 years). Specifically, they reported no significant differences in memory, attention and information processing capabilities between beetroot juice and placebo treatments.

The differences in cognitive performance results seen between these two studies could be attributed to methodological variations, including the duration of supplementation and the cognitive tests used. Gilchrist et al. [6] used a longer supplementation period (14 days) and included a simple reaction test; however, Kelly et al. [8] used a much shorter supplementation period (3 days) and did not include a simple reaction test. Additionally, participant selection may have impacted results as one study used older adults with type 2 diabetes [6], while the other used healthy older adults [8]. Therefore, the findings of this systematic review suggest that there may be some potential cognitive performance benefits for older adults following supplementation of dietary NO_3_^−^; however, these benefits may be specific to certain brain functions, and may be affected by the duration of supplementation as well as participant health status. Nevertheless, with only two studies to draw conclusions from, which provide conflicting results, more research is required to investigate these potential effects.

### 4.3. Nitrate Supplementation and Health

#### 4.3.1. Cardiovascular

Investigations into the possible cardiovascular health benefits of NO_3_^−^ supplementation in older adults has grown over the past 5 years, with a significant amount of research examining the effects on BP [2]; more recently, the effects on MAP [7], changes in blood flow [13], and endothelial function [16] have become additional areas of examination.

Due to age-related losses in NO production from decreased activity of NOS, older adults are at a higher risk for endothelial dysfunction and cardiovascular disease, of which, high BP and MAP are major risk factors [2]. Supplementation with dietary NO_3_^−^ increases NO production, which, in turn, has been shown to activate the enzyme, guanylate cyclase, which catalyses the conversion of guanosine triphosphate (GTP) to cyclic guanosine monophosphate (cGMP) [24]. Cyclic guanosine monophosphate acts on smooth muscle, causing relaxation, which results in vasodilation of arteries and veins, thus decreasing BP and reducing the risk of cardiovascular disease [8]. Despite this, the findings of this review indicate mixed results for the effects of acute and chronic dietary NO_3_^−^ supplementation on BP and MAP in older adults. Whilst five of the reviewed studies reported a reduction in SBP [5,7,8,12,13], with four of these also showing a reduction in DBP [5,7,8,13], a further five studies found no benefits [16,17,18,19,20]. Additionally, a more recent study found a reduction in MAP [7]; however, an earlier study showed no significant change [17].

The reasons for these conflicting results for BP may be due to differences in the health statuses of the participants. Of the five studies that reported a reduction in BP, only one used healthy older adults [8], while the others used older adults with COPD [5], CKD [7], HFpEF [12], or PAD [13]. Comparatively, three of the five studies which showed no significant change in BP, used healthy older adults [18,19,20], while one [16] used participants with risk factors for cardiovascular disease (not yet morbid), and one [17] used type 2 diabetic older adults. These differences could suggest that supplementation with dietary NO_3_^−^ may be more beneficial for reducing BP in older adults with morbidities closely related to an already reduced endothelial function, beyond that of age-related changes. Furthermore, the study by de Oliveira et al. [16] used a shorter absorption time of 2 h compared to the more common 2.5–4 h, used by studies which have shown significant reductions in BP [5,7]. This may not have provided sufficient time to allow for significant reductions in BP. In addition, Gilchrist et al. [17] investigated effects on BP while participants were taking antihypertensive and hypoglycaemic medication, which is likely to have impacted the results, as the participants’ baseline BPs would have already been lowered, due to the antihypertensive medication. However, one study has shown a decrease in BP in hypertensive patients, aged 18–85, following supplementation with NO_3_^−^-rich beetroot juice, while still adhering to their antihypertensive medication protocol, suggesting improvements may still be possible with supplementation in conjunction with drug protocols [26].

Alternatively, the conflicting results seen between these studies may have been due to differences in study design. Three of the studies which reported beneficial outcomes on BP in older adults following dietary NO_3_^−^ supplementation used prune juice [5], water [7] and orange juice [13] as the placebo controls, while all other studies [8,12,16,17,18,19,20], utilised a NO_3_^−^ depleted beetroot control, so there was potential for a ‘placebo’ effect. Also, as these three studies [5,7,13] utilised beetroot juice as the NO_3_^−^ source, but did not utilise a NO_3_^−^ depleted beetroot control, it is possible that other bioactive compounds found in beetroot juice (e.g., antioxidants and polyphenols) may have contributed to the reduction in BP [27]. However, other studies using a NO_3_^−^ rich beverage and NO_3_^−^ depleted beetroot juice (placebo) crossover design have still shown reductions in BP of older adults [8]. Furthermore, an investigation into the interruption of the NO_3_^−^–NO_2_^−^–NO pathway, via the use of mouthwash or spitting of saliva following beetroot juice consumption, has been shown to negate the reductive effects on BP, indicating high concentrations of NO_3_^−^ as the major contributor to a decreased BP [4]. The reasons for mixed results in MAP are likely similar to those mentioned above; including the continued use of participants’ antihypertension medications [17], or the use of an inappropriate study design (open-labelled with the placebo being water [7]).

In addition to reductions in BP and MAP, supplementation with dietary NO_3_^−^ in older adults has also been proposed to benefit cardiovascular health of older adults through its effects on blood flow and endothelial function [12]. Two studies found that acute supplementation with NO_3_^−^ rich beetroot gel (12.2 mmol NO_3_^−^, 2 h prior) [16] or juice, (9 mmol NO_3_^−^, 3 h prior) [13] improved endothelial function and blood flow in older adults. This was expected, as foods high in NO_3_^−^ have been shown to inhibit blood platelet aggregation and reverse or prevent further degradation in endothelial function [28], which is particularly important in older adults, as aging is associated with reduced blood flow and endothelial dysfunction [14]. Therefore, NO_3_^−^ supplementation plays an important role in maintaining NO homeostasis when endogenous production may be diminished, thus maintaining adequate blood flow and distribution [28]. Despite these results, Gilchrist et al. [17] found no significant differences in endothelial function following 14 days of NO_3_^−^ supplementation in older adults with type 2 diabetes, contradicting the aforementioned findings. As mentioned above, a major limitation in the study by Gilchrist et al. [17] was the continued use of participants’ antihypertensive medications, which are designed to promote vasodilation and subsequently increase blood flow, [2,4]. Therefore, it is unlikely that supplementation with dietary NO_3_^−^ would promote further benefits to result in improved endothelial function. Based on this, the lack of improvements in blood flow and endothelial function seen by Gilchrist et al. [17] are as expected.

Kemmner et al. [7] reported a reduced RRI in older adults with CKD following supplementation with 200 mL of NO_3_^−^ rich beetroot juice (~6 mmol/300 mg NO_3_^−^). Chronic kidney disease is another major risk factor for CVD, where hypertension can both result from, or be a large contributor to, the development of the disease [7]. Furthermore, a high RRI is associated with an increased risk of cardiovascular dysfunction [7]. Therefore, as older adults are at a greater risk of kidney dysfunction, due to age-related changes, a reduced RRI following supplementation with dietary NO_3_^−^ is likely to result in reduced risk of a cardiovascular event, thus improving cardiovascular health [7]. It is also important to note that CKD and high RRI can impact type 2 diabetes, potentially affecting metabolic heath as well.

Despite the mixed results reported for changes in the BP and MAP of older adults following dietary NO_3_^−^ supplementation, the review of the published data indicates that there is potential for beneficial effects which can result in improved cardiovascular health. Furthermore, some evidence from the reviewed studies also indicates potential benefits to cardiovascular health of older adults, via improved blood flow, endothelial function and/or RRI post-supplementation with dietary NO_3_^−^. Therefore, the conflicting results warrant further research, to fully evaluate the extent to which dietary NO_3_^−^ supplementation impacts cardiovascular health in older adults. Additionally, specific attention should be drawn to participant health status and the use of a double-blind study design and NO_3_^−^ depleted control.

#### 4.3.2. Cerebrovascular

Only one study [14] met the inclusion criteria for examining the relationship between dietary NO_3_^−^ consumption and cerebrovascular health. Presley et al. [14] showed increased perfusion in the frontal cortex of the brain (using MRI), of older adults following a diet high in NO_3_^−^ with additional beetroot juice supplementation. This suggests that dietary NO_3_^−^ supplementation may attenuate the age-related drop in NO activity and loss of cerebral blood flow (a major contributor to neurogenerative diseases) [5], thus potentially slowing or even preventing the development of cerebrovascular dysfunction. However, with only one study to draw conclusions from, cause and effect cannot be confirmed. Additionally, no study to date has directly investigated the long-term effects of dietary NO_3_^−^ supplementation on the development of neurodegenerative diseases. Therefore, further research is required to investigate the effects of dietary NO_3_^−^ supplementation on specific diseases of the brain and determine its possible benefits to cerebrovascular health in older adults.

#### 4.3.3. Metabolic

The current systematic review found two studies which examined the relationship between metabolic health and dietary NO_3_^−^ supplementation in older adults, both focusing predominately on insulin sensitivity and changes in plasma glucose levels [17,19]. Nitric oxide helps shuttle glucose absorbed in the small intestine into skeletal muscle [29]. A reduction in NO bioavailability has been shown to result in a decrease in insulin secretion and reduced activation of GLUT4 transporter proteins, and thus a subsequent drop in glucose uptake [19,27]. Therefore, older adults may have an age-related drop in insulin sensitivity, due to a reduction in NO production [17]. Therefore, supplementation with dietary NO_3_^−^ in older adults may improve insulin sensitivity, improving metabolic health. However, the results from the studies included in this review do not support this suggestion. Neither acute supplementation of NO_3_^−^ rich beetroot juice (140 mL, ~11.91 mmol/600 mg NO_3_^−^) in healthy older adults [19] nor chronic supplementation (250 mL·day^−1^, ~7.5 mmol/400 mg NO_3_^−^) in type 2 diabetic older adults [17] resulted in significant differences in plasma glucose levels or insulin sensitivity. These results suggest that dietary NO_3_^−^ supplementation in older adults does not affect metabolic health via improvements in insulin sensitivity. However, it is important to note that there were some limitations to these studies. Gilchrist et al. [17] had participants continue their prescribed hypoglycaemic medications, which are designed to increase insulin secretion or improve insulin action [30], thereby potentially masking any potential metabolic effects of dietary NO_3_^−^ supplementation. Additionally, Shepherd et al. [19] used healthy older adults with a younger mean age (59.2 ± 6 years) compared to other studies, which may have impacted results, as age-related NO dysfunction may not become an issue until individuals are in their mid-60 s [4], thus, the potential benefits would not be as great in the early stages of older adulthood. With only two studies examining the metabolic health benefits of dietary NO_3_^−^ supplementation in older adults, further investigation is required to provide a stronger evidence base, to allow more in-depth analyses and conclusions to be made.

Certain limitations for this systematic review should be noted. Firstly, there was incomplete retrieval of identified research, as only English articles were included in this systematic review. Secondly, studies were generally characterised by small sample sizes and there were few studies investigating some outcome variables, which may have affected the reliability of the conclusions drawn in this systematic review. A third limitation to our analysis was the lack of a meta-analysis. Due to the small number of studies for each outcome variable, completing a meta-analysis would have resulted in weak representation of the data, thus, it is suggested that a meta-analysis be conducted in the future, once more studies have been published. This would be beneficial because it would provide greater statistical evidence on the effects of dietary NO_3_^−^ supplementation in older adults.

## 5. Conclusions

In summary, the findings of this systematic review provide convincing evidence for improved physiological performance (increased time to fatigue), in older adults following dietary NO_3_^−^ supplementation. Some evidence suggests positive outcomes of dietary NO_3_^−^ supplementation on cardiovascular health (reduced BP and MAP, increased blood flow and improved endothelial function, and reduced RRI) and cerebrovascular health (increased CBF), while results are mixed as to whether dietary NO_3_^−^ supplementation has benefits on cognitive performance. Metabolic health in older adults does not seem to be improved via changes in insulin sensitivity or plasma glucose levels following dietary NO_3_^−^ supplementation; however, more research is required before firm conclusions can be made. Furthermore, it is evident that these effects may be influenced by the dosage of NO_3_^−^, duration of supplementation, blinding/placebo-control, and health status of participants. Therefore, further research is required in this area, with a focus on randomised, double-blind, NO_3_^−^ depleted control-based studies, with particular attention to participant health status.

## Figures and Tables

**Table 1:** Summary of studies investigating the effects of dietary nitrate supplementation on physical performance, cognitive performance, and health-based outcomes in older adults.

Reference	Participants	Study Design	Supplementation Protocol	Testing Protocols	Blood Measures (NO Indices)	Physiological Performance Outcomes	Cognitive Performance Outcomes	Health-Based Outcomes
Berry et al., 2015 [5]	15 COPD patients (m/f 12/3) (age 69.6 ± 8.5)	Randomised, single-blind crossover	Acute supplementation, 2.5 h prior to exercise, 140 mL BR (7.58 mmol NO_3_]) (Note PL used was prune juice)	Constant work rate test at 75% max on cycle ergometer	↑ Plasma [NO_2_^−^] ↑ Plasma [NO_3_^−^]	↑ Time to exhaustion during submaximal cycle exercise	NT	↓ SBP ↓ DBP
Eggebeen et al., 2016 [12]	20 HFpEF patients (m/f 3/17) (age 69 ± 7)	Randomised, double-blind crossover	Phase 1: Acute supplementation, 1.5–2 h prior to exercise, 70 mL BR (6.1 mmol NO_3_^−^) Phase 2: 7 days supplementation, 70 mL·day^−1^ BR (6.1 mmol·day^−1^ NO_3_^−^)	Constant work rate test at 75% max on cycle ergometer	↑ Plasma [NO_2_^−^] ↑ Plasma [NO_3_^−^]	↑ Time to exhaustion during submaximal cycle exercise	NT	↓ SBP
Kenjale et al., 2011 [13]	4 male and 4 female PAD patients (age 67 ± 13)	Randomised, open-label, crossover	Acute supplementation, 3 h prior to exercise, 500 mL BR (9 mmol NO_3_^−^) (Note: orange juice was used as PL)	Walking maximal cardiopulmonary exercise (CPX) test	↑ Plasma [NO_2_^−^] ↑ Plasma [NO_3_^−^]	↑ Walking duration longer before onset of pain (18%) ↑ Maximum walking time (17%)	NT	↑ Tissue blood flow (NIRS) ↓ DBP ↓ SBP and HR during recovery
Siervo et al., 2016 [20]	19 healthy older adults (m/f 9/10) (age 64.7 ± 3)	Randomised, double-blind crossover	7 days supplementation, 2 × 70 mL·day^−1^ BR (12 mmol·day^−1^ NO_3_^−^)	Incremental cycle test to voluntary exhaustionHand-grip strength testTime up-and-go testRepeated chair raising test10-m walking test	↑ Plasma [NO_3_^−^] ↑ Urinary [NO_3_^−^]	↔ O_2_ consumption ↔ Hand-grip strength ↔ Up-and-go test ↔ Repeated chair raising test ↔ 10-m walking test	NT	↔ BP
Kelly et al., 2013 [8]	6 male and 6 female older adults (age 64 ± 4; 63 ± 2)	Randomised, double-blind crossover	3 days supplementation, 140 mL·day^−1^ BR (9.6 mmol·day^−1^ NO_3_^−^)	Walking treadmill test and leg ergometer. Cognitive function tests	↑ Plasma [NO_2_^−^]	↓$\dot{V}O_{2}$ response time from rest to walking ↔ 6 m walking test	↔ Cognitive tests	↓ SBP ↓ DBP
Gilchrist et al., 2014 [6,12]	27 older adults with type 2 diabetes (m/f 18/9) (age 67.2 ± 4.9)	Randomised, double-blind crossover	14 days supplementation, 250 mL·day^−1^ BR (7.5 mmol NO_3_^−^)	5 cognitive tests on E-Studio software	↑ Plasma [NO_2_^−^] ↑ Plasma [NO_3_^−^]	NT	↓ Simple reaction time (improvement)	NT
Presley et al., 2011 [14]	14 healthy older adults (sex not stated) (age 74.7 ± 6.9)	Placebo-controlled, crossover	2 days high NO_3_^−^ diet with BR (~12.4 mmol NO_3_^−^) Low NO_3_^−^ diet (~0.089 mmol NO_3_^−^)	NT	↑ Plasma [NO_2_^−^] ↑ Plasma [NO_3_^−^]	NT	NT	↑ Cerebral perfusion (MRI)
de Oliveira et al., 2016 [16,17]	20 older adults with risk factors for cardiovascular disease (m/f 7/13) (age 70.5 ± 5.6)	Randomised, double-blind crossover	Acute supplementation, 2 h prior to measurements, 100 g BG (12.2 mmol NO_3_^−^) (PL used was NO_3_^−^ depleted gel)	NT	↑ Urinary [NO_2_^−^] ↑ Urinary [NO_3_^−^]	NT	NT	↑ Endothelial function ↔ BP ↔ Arterial stiffness
Gilchrist et al., 2013 [17]	27 older adults with type 2 diabetes (m/f 18/9) (age 67.2 ± 4.9)	Randomised, double-blind crossover	14 days supplementation, 250 mL·day^−1^ BR (7.5 mmol NO_3_^−^)	NT	↑ Plasma [NO_2_^−^] ↑ Plasma [NO_3_^−^]	NT	NT	↔ BP ↔ MAP ↔ Endothelial function ↔ Insulin sensitivity
Kemmner et al., 2017 [7]	17 older adults with CKD (m/f 7/10) (age 72 ± 6)	Randomised, open-label crossover	Acute supplementation, 4 h prior to repeat measures, 200 mL BR (300 mg NO_3_^−^) (Note PL used was water)	Hemodynamic parameters and renal resistance index measure	↑ Plasma [NO_3_^−^]	NT	NT	↓ SBP ↓ DBP ↓ MAP ↓ RRI
Miller et al., 2012 [18]	8 normotensive older adults (m/f 3/5) (age 72.5 ± 4.7)	4-arm, randomised, crossover	3 days low NO_3_^−^ diet (0.70 mmol NO_3_^−^) Low NO_3_^−^ diet + supplementation, 500 mL BR (8.5 mmol NO_3_^−^) High NO_3_^−^ diet (2.50 mmol NO_3_^−^) High NO_3_^−^ diet + supplementation 500 mL BR	NT	↑ Plasma [NO_2_^−^] ↑ Plasma [NO_3_^−^]	NT	NT	↔ BP
Shepherd et al., 2016 [19]	15 healthy older adults (m/f 8/7) (age 59.2 ± 6)	Randomised, double-blind crossover	Acute supplementation, 140 mL BR (11.91 mmol [NO_3_^−^])	NT	↑ Plasma [NO_2_^−^] ↑ Plasma [NO_3_^−^]	NT	NT	↔ BP ↔ Hepatic blood flow (MRI) ↔ Plasma glucose ↔ C-peptide ↔ [Incretin]

All values are mean ± SD. ↑ significant increase;↓ significant decrease; ↔ no significant difference; m/f, males/females; NT, not tested; [NO_2_^−^], nitrite concentration; [NO_3_^−^], nitrate concentration; BR, beetroot juice; PL, placebo; BG, beetroot gel; BP, blood pressure; SBP, systolic blood pressure; DBP, diastolic blood pressure; MAP, mean arterial pressure; RRI, renal resistive index; $\dot{V}O_{2}$, oxygen uptake; HR, heart rate; NIRS, near infrared spectroscopy; MRI, magnetic resonance imaging; COPD, chronic obstructive pulmonary disease; HFpEF, heart failure with preserved ejection function; CKD, chronic kidney disease; PAD, peripheral arterial disease.
